# Clinical features and prognosis of MPO-ANCA and anti-GBM double-seropositive patients

**DOI:** 10.3389/fimmu.2022.991469

**Published:** 2022-10-27

**Authors:** Xueling Hu, Chanjuan Shen, Ting Meng, Joshua D. Ooi, Peter J. Eggenhuizen, Ya-ou Zhou, Hui Luo, Jin-biao Chen, Wei Lin, Yizi Gong, Qi Xiong, Jia Xu, Ning Liu, Xiangcheng Xiao, Rong Tang, Yong Zhong

**Affiliations:** ^1^ Department of Nephrology, Xiangya Hospital, Central South University, Changsha, China; ^2^ Key Laboratory of Biological Nanotechnology of National Health Commission, Xiangya Hospital, Central South University, Changsha, China; ^3^ Department of Hematology, The Affiliated Zhuzhou Hospital of Xiangya Medical College, Central South University, Zhuzhou, China; ^4^ Centre for Inflammatory Diseases, Department of Medicine, Monash Medical Centre, Monash University, Clayton, VIC, Australia; ^5^ Department of Rheumatology and Immunology, Xiangya Hospital, Central South University, Changsha, China; ^6^ Department of Medical Records and Information, Xiangya Hospital, Central South University, Changsha, China; ^7^ Department of Pathology, Xiangya Hospital, Central South University, Changsha, China

**Keywords:** ANCA-associated vasculitis, renal survival, myeloperoxidase, anti-GBM disease, double positive vasculitis, risk factors

## Abstract

**Background:**

Several lines of evidence implicate that there are distinct differences between patients with myeloperoxidase (MPO)-antineutrophil cytoplasmic antibody (ANCA) and anti-glomerular basement membrane (GBM) antibody double-seropositive patients (DPPs) and single-positive patients. Hence, we conducted a retrospective study from a single center in China to analyze the clinical and pathological features, and prognosis of DPPs.

**Methods:**

109 patients with MPO-ANCA-associated vasculitis (MPO-AAV), 20 DPPs and 23 patients diagnosed with anti-GBM disease from a large center in China were included in this study. The ratio of patients with renal biopsy in three groups were 100%, 50% and 100%, respectively. Their clinical and pathological characteristics, and outcomes were analyzed. The intensity of immune deposits in the kidney at diagnosis was detected by immunofluorescence (IF). Furthermore, multivariate Cox hazard model analysis was used to assess the clinical and histological predictors of end-stage renal disease (ESRD) and death for DPPs.

**Results:**

In our study, we found that patients in the DPPs group were older than the other two groups (*p* = 0.007, MPO-AAV vs. DPPs; *p* < 0.001, DPPs vs. anti-GBM). The DPPs group had a higher value of serum creatinine (*p* = 0.041) and lower estimated glomerular filtration rate (eGFR) (*p* = 0.032) compared with MPO-AAV patients. On the contrary, the DPPs group had a lower serum creatinine (*p* = 0.003) compared with patients with anti-GBM group. The proportion of patients with cardiac system involvement in the DPPs group was higher than anti-GBM patients (*p* = 0.014). Cellular crescents could be generally observed in renal biopsy of DPPs and patients with anti-GBM glomerulonephritis. In addition, Bowman’s capsule rupture was more common in DPPs than MPO-AAV patients (*p* = 0.001). MPO-AAV had a better renal and overall survival outcome than DPPs (*p* < 0.001). There was no significant difference of renal and overall survival outcome between DPPs and patients with anti-GBM disease. The incidence of ESRD in DPPs was negatively associated with lymphocyte count (HR 0.153, 95% CI 0.027 to 0.872, *p* = 0.034) and eGFR (HR 0.847, 95% CI 0.726 to 0.989, *p* = 0.036). Elevated serum creatinine was confirmed as a risk factor of both renal (HR 1.003, 95% CI 1.000 to 1.005, *p* = 0.019) and patient survival in DPPs (HR1.461, 95% CI 1.050 to 2.033, *p* = 0.024).

**Conclusion:**

In summary, compared with anti-GBM disease, DPPs tended to involve multi-organ damage rather than limited to the kidney. It is highlighted that serologic DPPs have a worse renal and patient prognosis than MPO-AAV. Moreover, we found that the risk factors of renal survival of DPPs include low lymphocyte count, elevated serum creatinine and reduced eGFR, and serum creatinine can predict patient survival.

## Introduction

ANCA-associated vasculitis (AAV) is a life-threatening disease characterized by necrotic inflammation of small and medium-sized blood vessels (1). Accumulating evidence support that MPO-AAV and proteinase 3 (PR3)-AAV are distinct diseases, as we reported previously, and the former constituted the majority in China (2). The detection of serologic anti-GBM positivity in AAV patients is not uncommon (3). The prevalence of double-seropositive patients (DPPs) has not been ascertained in Chinese. Previous studies suggested that up to 47% of patients with anti-GBM disease were also positive for ANCA and 5-14% of patients with AAV were simultaneously positive for anti-GBM antibody (4–6). There is controversy as to the prognosis of patients with DPPs (7). In addition, it is unclear at present whether histological lesions can predict the incidence of ESRD or death in DPPs. Therefore, the clinical and pathological features of DPPs in our center were comprehensively analyzed. Moreover, the potential clinical and pathological predictors of renal prognosis for ESRD and death in DPPs were also explored (8, 9).

## Methods

### Patients and data collection

A single-center retrospective analysis was performed based on 152 patients who attended in Xiangya Hospital of Central South University from January 1, 2010 to June 30, 2021. There were three groups classified by the presence of MPO-ANCA and anti-GBM as follows: 1) patients with single-positivity of MPO-ANCA; 2) patients with double positivity of MPO-ANCA and anti-GBM; 3) patients with single- positivity of anti-GBM.

The patients with a diagnosis of MPO-AAV all conformed to the 2012 Chapel Hill Consensus Conferences Nomenclature of Vasculitis and circulating MPO-ANCA can be recognized by antigen-specific enzyme-linked immunosorbent assay (ELISA). Those affected by viral infections such as hepatitis B or C and who had lupus nephritis, IgA nephropathy or other systemic autoimmune disorders were excluded. Those confirmed as MPO-AAV patients with anti-GBM positivity were diagnosed as DPPs in our study. When either of the following two indicators were found, anti-GBM disease was diagnosed: 1) serological evidence for anti-GBM antibodies in the peripheral blood responsible for severe alveolar hemorrhage and/or rapidly progressive glomerulonephritis (RPGN); 2) the deposition of linear immunoglobulin G along the GBM seen in the renal specimen (10, 11).

Informed consent was obtained from each included patient. All the baseline data were reviewed from the electronic medical record system in the hospital. Details associated with outcomes in the natural course of disease were also collected. Patients were followed up from the time of initial diagnosis to the occurrence of ESRD or death or the deadline of follow-up. ESRD was defined as eGFR less than 15 ml/min*1.73 m^2^ or maintenance of renal replacement therapy (RRT) for more than 3 months or kidney transplantation. eGFR was calculated based on the Chronic Kidney Disease Epidemiology Collaboration (CKD-EPI) equation.

### Renal biopsy

142 out of the 152 (93%) patients underwent kidney biopsy at presentation prior to commencement of immunosuppressive drugs. There were 109, 10 and 23 patients receiving kidney biopsy in MPO-AAV group, DPPs group and anti-GBM group, respectively. Biopsies were scored by two pathologists with an optical microscope and they were blinded to the clinical condition to ensure objectivity. The number of integral glomeruli was more than or equal to ten in all specimens reaching the minimum required value. The proportions of cellular crescent and global glomerulosclerosis were calculated. In addition, histological features including fibroid necrosis, granuloma-like lesions, Bowman’s capsule rupture, thrombotic microangiopathy (TMA), and the scores of interstitial infiltration and tubulointerstitial injury were all evaluated. Interstitial infiltrate and tubulointerstitial lesions were graded semi-quantitatively according to the level of involvement (scale 0-3: score 0 for normal, score of 1 for < 25%, 2 for 25-50%, and 3 for > 50%) (9).

### Immunofluorescence staining and Immunohistochemistry

As we reported previously (12), both indirect immunofluorescence (IF) assay for p-ANCA or c-ANCA (Werfen, 708298) and ELISA for MPO-ANCA (Werfen, 704655) and PR3-ANCA (Werfen, 704660) in all patients were performed to test serum ANCA levels. Antigen-specific ELISAs were also performed to determine the circulating anti-GBM antibody level (Werfen, 708740). By staining with fluorescein-conjugated (FITC) antisera specific for human IgA, IgM, IgG, C3, C1q and C4, their intensity in the renal biopsies can be scored under an immunofluorescence microscope (9). Scale 0-3: 0 for negative (–), 1 for trace (±) and mild (1+), 2 for moderate (++) and 3 for strong (+++).

### Statistical analysis

The raw data of patients involving demographics, laboratorial index and histological changes as well as subsequent outcomes were compared between the three groups. Mean ± SD or percentage was employed to describe normally distributed variables whereas median or interquartile range (IQR) was applied to express non-normally distributed variables. ANOVA with *post hoc* LSD test was used to compare significant differences in continuous variables among groups. Differences in categorical variables were analyzed by chi squared test or Fisher’s exact test. Meanwhile, significant differences of renal survival and patient survival were determined by Kaplan–Meier curves using log-rank test. With the aim of identifying relevant clinical and pathological factors which could predict ESRD or death, we carried out multivariate cox regression analysis and determined hazard ratios (HRs) with 95% confidence intervals (95% CIs). A *p* value < 0.05 was used as the selection criterion. Backward elimination was undertaken to filter out significant predictor variables. All data analyses were performed in SPSS statistical software (version 26.0).

## Results

### Conventional clinical characteristics

Altogether, 152 patients in our cohort were assigned to three groups based on the type of serum antibodies. There were 109 patients in the MPO-AAV group, concurrently 20 in DPPs group and 23 in anti-GBM group. The MPO-ANCA concentration of all patients diagnosed with AAV in our study was detected by ELISA with an average level of 76.67 (40.79,121.86) U/ml in the MPO-AAV group whereas the average level was 96.02 ± 12.49 U/ml in the DPPs group. All the demographic and clinical features were presented in **Table 1**. Compared with the other two groups, the patients in the DPPs group were older with an average age of 64.50 ± 9.77 years, and the patients in the anti-GBM group were the youngest (*p* < 0.05, MPO-AAV vs. DPPs; *p* < 0.05, DPPs vs. anti-GBM). No distinct difference in gender ratio was found among three groups. Our data showed that patients in three groups generally had moderate anemia yet without obvious statistical differences. Both the serum albumin and globulin in the anti-GBM group were detected at relatively lower levels than the other two groups (*p* < 0.05, anti-GBM vs. MPO-AAV; *p* < 0.05, anti-GBM vs. DPPs), and the mean value of serum albumin was less than 30g/L. It was found that the level of serum creatinine among three groups widely varied between each other. The anti-GBM group displayed the highest level of serum creatinine, and the creatinine value of DPPs was higher than that of MPO-AAV alone. Correspondingly, the level of eGFR of the MPO-AAV group was higher than that of other two groups, while the levels of eGFR of the DPPs group and anti-GBM group were similar. Concerning serum immunological data, it was noteworthy that we also found a lower level of circulating IgG in the anti-GBM group compared with the other two groups (p < 0.05, anti-GBM vs. MPO-AAV; p < 0.05, anti-GBM vs. DPPs). Kidney involvement was observed in all subjects, which manifested as hematuria, proteinuria, oliguria or anuria, hypertension and edema, which led to a rapid decline in kidney function. Interestingly, we found that patients in the DPPs group tended to have cardiac system involvement.

**Table 1:** Baseline clinical characteristics.

	MPO-AAV (n = 109)		DPPs (n = 20)	Anti-GBM (n = 23)	*p* value
Age (year) (mean, SD)	54.17 ± 16.37		64.50 ± 9.77^a^	41.78 ± 14.55^a,b^	<0.0001
Sex (male/female)	58/51		12/8	3/10	0.842
Neutrophil (10^9^/L) (mean, SD)	7.03 ± 4.62		6.66 ± 2.10	7.29 ± 3.85	0.887
Lymphocyte (10^9^/L) (mean, SD)	1.45 ± 1.55		0.99 ± 0.40	1.31 ± 0.68	0.365
Hemoglobin (g/L) (mean, SD)	81.61 ± 17.20		77.85 ± 10.38	81.04 ± 19.55	0.659
Platelet (10^9^/L) (mean, SD)	256.38 ± 106.57		262.60 ± 115.88	252.52 ± 106.51	0.953
Serum albumin (g/L) (mean, SD)	39.71 ± 14.24		37.31 ± 14.67	27.57 ± 5.05^a,b^	0.001
Serum globulin (g/L) (mean, SD)	31.97 ± 6.63		32.88 ± 7.42	27.76 ± 6.88^a,b^	0.017
Urinary protein (g/24 h) (median, IQR)	1.53 (0.85,3.70)		1.03 (0.50,3.42)	3.06 (1.26,5.00)	0.17
Serum creatinine (μmol/L) (mean, SD)	374.88 ± 251.15		514.53 ± 241.32^a^	826.29 ± 403.70^a,b^	<0.0001
eGFR (ml/min per 1.73 m^2^) (median, IQR)	18.02 (8.50,27.77)		8.58 (6.60,16.90)^a^	5.39 (3.82,13.13)^a^	<0.0001
CRP (mg/dL) (median, IQR)	16.20 (4.99,41.80)		24.20 (9.88,89.30)	31.75 (5.86,110.00)	0.113
ESR (mm/h) (median, IQR)	71 (42,110)		86 (35,120)	41 (20,100)	0.093
Serum immunological indexes					
sC3 (mg/L) (mean, SD)	819.92 ± 243.45	807.20 ± 190.96		896.09 ± 273.21	0.357
sC4 (mg/L) (mean, SD)	264.65 ± 110.07	225.87 ± 57.04		277.65 ± 96.20	0.218
sIgA (mg/L) (mean, SD)	2674.11 ± 1407.03	2229.05 ± 1017.77		2229.87 ± 1378.32	0.198
sIgG (g/L) (mean, SD)	14.67 ± 4.79	14.80 ± 5.75		9.49 ± 4.63^a,b^	<0.0001
sIgM (mg/L)(mean, SD)	1121.20 ± 659.87	1056.35 ± 350.08		975.00 ± 531.31	0.562
Organ involvement					
Kidney, n%	109 (100.00)	20 (100.00)		23 (100.00)	1.0000
Pulmonary, n%	60 (55.05)	15 (75.00)		10 (43.48)	0.111
Cardiovascular, n%	14 (12.84)	6 (30.00)		0 (0.00)^b^	0.014
Nervous system, n%	13 (11.93)	3 (15.00)		0 (0.00)	0.139
BVAS (mean, SD)	16.31 ± 5.75	16.55 ± 5.78		–	0.865
ESRD, n (%)	37 (33.94)	15 (75.00)^a^		19 (82.61)^a^	<0.0001
Mortality, n (%)	9 (8.26)	6 (30.00)^a^		2 (8.70)	0.001

eGFR, estimated glomerular filtration rate; CRP, C-reactive protein; ESR, erythrocyte sedimentation rate; BVAS, birmingham vasculitis activity score; ESRD, end stage renal disease;

a p < 0.05 vs. MPO-AAV and ^b^p < 0.05 vs. DPPs.

Furthermore, all data from the patients who received a renal biopsy were also comprehensively analyzed. The results regarding age, eGFR, serum IgG level and cardiovascular involvement, as shown in **Supplementary Table 1**, were in accordance with those in **Table 1**.

### Pathological index

Renal biopsies were performed in all patients except 10 patients in the DPPs group. Histological characteristics and immunofluorescence patterns of these 142 patients were evaluated (**Table 2**). About 57% of involved glomeruli in the DDPs group were crescents, and approximately 66% of these were cellular crescents, which is markedly different from the MPO-AAV group (*p* = 0.006). In the anti-GBM group, crescent formation was observed in 77% of the affected glomeruli, of which 50% were cellular crescents. Of note, we found that the proportion of glomerular fibrinoid necrosis in the DPPs group had a higher trend compared with the other two groups, although the difference did not reach statistical significance.

**Table 2:** Pathological features.

	MPO-AAV (n=109)	DPPs (n=10)	Anti-GBM (n=23)	*p* value
Histological characteristics				
The proportion of Glomerular sclerosis (median, IQR)	0.28 (0.13,0.50)	0.14 (0.03,0.42)	0.13 (0.00,0.25)	0.01
The proportion of crescentic glomeruli (median, IQR)	0.38 (0.16,0.56)	0.57 (0.51,0.77)^a^	0.77 (0.47,0.89)^a^	<0.0001
The proportion of cellular crescents (median, IQR)	0.00 (0.00,0.37)	0.66 (0.17,1.00)^a^	0.50 (0.00,1.00)^a^	<0.0001
The proportion of fibrous crescents (median, IQR)	0.07 (0.00,0.40)	0.00 (0.00,0.14)	0.00 (0.00,0.25)	0.174
Fibrinoid necrosis, n%	44 (40.4)	7 (70.0)	13 (56.5)	0.098
Bowman’s capsule rupture, n%	36 (33.0)	9 (90.0)^a^	15 (65.2)^a^	<0.0001
Granulomatous lesions, n%	9 (8.3)	1 (10.0)	2 (8.7)	1.000
TMA, n%	9 (8.3)	1 (10.0)	1 (4.3)	0.865
Interstitial infiltrates (n, 0/1/2/3)	(0/49/50/10)	(0/3/3/4)	(0/10/12/1)	0.094
Tubulointerstitial lesions (n, 0/1/2/3)	(0/47/45/17)	(0/3/4/3)	(0/11/11/1)	0.382
Immunofluorescence pattern (n%)				
C3 Number of negative	75 (69.4)	6 (60.0)	6 (26.1)	
Number of 1+	17 (15.7)	2 (20.0)	4 (17.4)^a,b^	<0.0001
Number ≥ 2+	16 (14.8)	2 (20.0)	13 (56.5)	
C4 Number of negative	83 (86.5)	9 (90.0)	3 (30.0)	
Number of 1+	11 (11.5)	0 (0.0)	3 (30.0)^a,b^	<0.0001
Number ≥ 2+	2 (2.1)	1 (10.0)	4 (40.0)	
C1q Number of negative	95 (88.8)	10 (100.0)	20 (90.9)	
Number of 1+	9 (8.4)	0 (0.0)	2 (9.1)	1.000
Number ≥ 2+	3 (2.8)	0 (0.0)	0 (0.0)	
IgA Number of negative	85 (81.0)	9 (90.0)	13 (56.5)	
Number of 1+	17 (15.9)	0 (0.0)	6 (26.1)^a^	0.032
Number ≥ 2+	3 (2.9)	1 (10.0)	4 (17.4)	
IgG Number of negative	80 (74.1)	5 (50.0)	5 (21.7)	
Number of 1+	16 (14.8)	2 (20.0)	4 (17.4)^a^	<0.0001
Number ≥ 2+	12 (11.1)	3 (30.0)	14 (60.9)	
IgM Number of negative	58 (53.7)	4 (40.0)	11 (47.8)	
Number of 1+	23 (21.3)	2 (20.0)	5 (21.7)	0.555
Number ≥ 2+	27 (25)	4 (40.0)	7 (30.4)	

TMA, thrombotic microangiopathy; ^a^p < 0.05 vs. MPO-AAV; ^b^p < 0.05 vs. DPPs.

Interestingly, Bowman’s capsule rupture could be observed in 90% renal specimens in the DPPs group, which was the highest proportion among three groups. According to our data, deposition of C3, C4, IgA and IgG in the DPPs group trended toward being higher compared with the MPO-AAV group. However, no significant difference in the distribution of both complement and immunoglobulin between MPO-AAV and DPPs groups was found. Unlike the anti-GBM group, the other two groups were more consistent with the oligo-immune complex deposition type.

### Regimen

As presented in **Supplementary Table 2**, most patients in the DPPs group received the standard induction therapy including corticosteroids combined with cyclophosphamide similar to patients in the MPO-AAV group and the anti-GBM group. With regard to the treatment of plasma exchange, there was a significant difference between the MPO-AAV group and DPPs group (*p* < 0.05). Only 34 MPO-AAV patients (31.2%) in our cohort were treated with plasma exchange while 13 (65.0%) of DPPs and 18 (78.3%) patients with anti-GBM disease received plasma exchange. In addition, some patients received intravenous pulses of methylprednisolone at diagnosis.

### Survival analysis and risk factor

**Figure 1:**
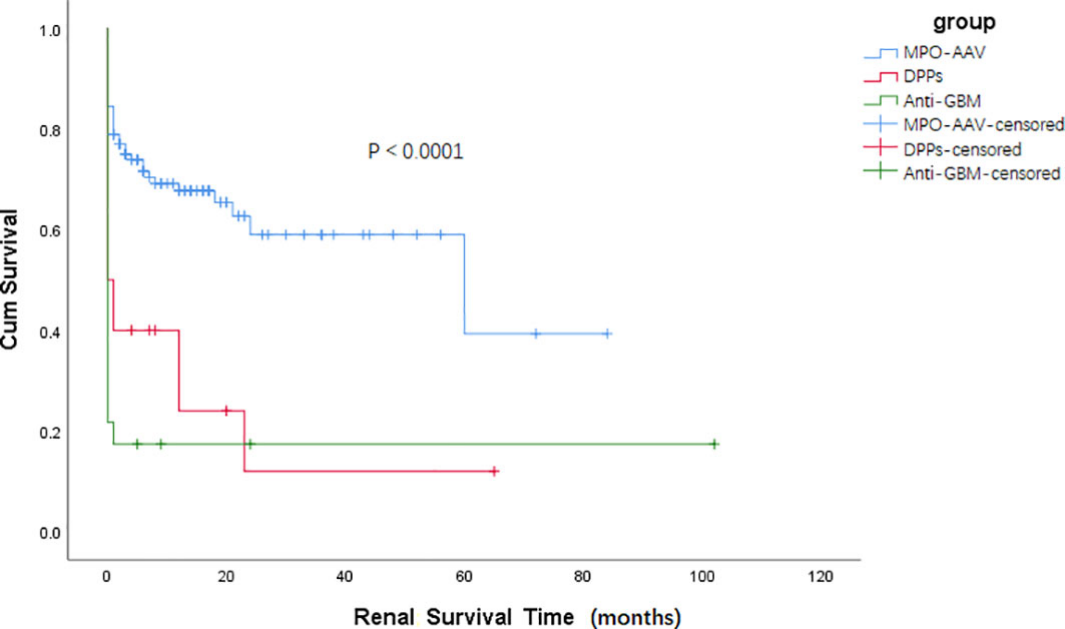
Kaplan-Meier survival functions describing long-term renal survival states of three groups Renal survival time (months) refers to the time since the start of treatment p < 0.001, MPO-AAV vs DPPs; p < 0.0001, MPO-AAV vs. Anti-GBM; p = 0.307, DPPs vs. Anti-GBM.

**Figure 2:**
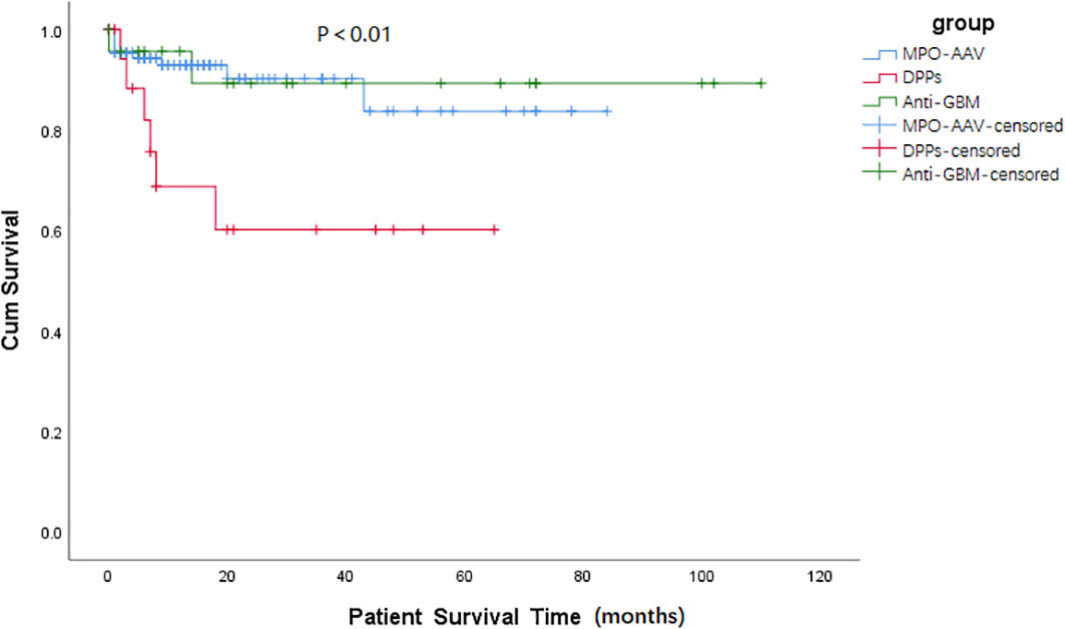
Kaplan-Meier survival functions describing long-term patient survival states of three groups. Patient survival time (months) refers to the time since the start of treatment. p = 0.853, MPO-AAV vs. Anti-GBM; p = 0.006, MPO-AAV vs. DPPs; p = 0.055, DPPs vs. Anti-GBM.

Results about renal and patient survival have been demonstrated in **Figures 1**, **2**. Similar to patients in the anti-GBM group, patients in the DPPs group had a worse renal prognosis when compared with patients in the MPO-AAV group (*p* < 0.001, **Figure 1**). In the DPPs group, 75% of cases finally progressed to ESRD during follow-up, with a medium survival time of 12.58 (1.47, 23.69) months. Regarding patient survival, patients in the DPPs group had a worse prognosis compared with MPO-AAV patients (*p* < 0.01, **Figure 2**). The details for outcomes of patients in three groups with a renal biopsy were displayed in **Supplementary Figure 1**. Consistently, our results revealed that patients in the DPPs group who underwent renal biopsy had a worse renal prognosis when compared with patients in the MPO-AAV group (*p* = 0.001, **Supplementary Figure 1**). However, there was no significant difference between the MPO-AAV group and the DPPs group with a renal biopsy regarding patient survival (*p* = 0.231, **Supplementary Figure 2**).

According to multivariate Cox analysis (**Table 3**), low lymphocyte count (HR 0.153, 95% CI 0.027 to 0.872, *p* = 0.034), elevated serum creatinine (HR 1.003, 95% CI 1.000 to 1.005, p = 0.019) and reduced eGFR (HR 0.847, 95% CI 0.726 to 0.989, *p* = 0.036) were independent predictors for ESRD in patients with DPPs. Serum creatine was shown to be an independent risk factor for death of DPPs (HR 1.461, 95% CI 1.050 to 2.033, *p* = 0.024, **Table 4**).

**Table 3:** Multivariable predictors of ^a^ESRD in DPPs by multivariate COX regression analysis.

Predictors	*p* value	HR^b^ (95%CI^c^)
Leucocyte count	0.034	0.153 (0.027-0.872)
Serum creatinine	0.019	1.003 (1.000-1.085)
eGFR	0.036	0.847 (0.726-0.989)

a ESRD, end stage renal disease; ^b^HR, hazard ratio; ^c^CI, confidence interval.

**Table 4:** Multivariable predictors of death in DPPs by multivariate COX regression analysis.

Predictors	*p* value	HR^a^ (95%CI^b^)
Serum creatinine	0.024	1.461 (1.050-2.033)
BUN	0.094	1.062 (0.990-1.138)
eGFR	0.092	0.720 (0.491-1.056)

a HR, hazard ratio; ^b^CI, confidence interval.

## Discussion

Serological detection of anti-GBM antibodies in MPO-AAV patients is not uncommon (13, 14). The scientific community had put forward many hypotheses about the two antibodies, although the mechanism of action between them has not been clearly studied. Several studies suggested that AAV might develop first following its natural disease history, and then anti-GBM antibodies could emerge with corresponding clinical manifestation (15–18). It warrants further study to explore the differences between isolated MPO-ANCA or anti-GBM positive and double positive types, including original serological, immunological, pathological and prognostic index.

In this retrospective study, older age, slightly higher albumin and globulin level, intermediate level of serum creatinine and similar serum IgG value with MPO-AAV could be found in the DPPs group compared with other two sets. As early as 2005, Rutgers et al. have reported that the average age of the affected population was older in MPO-AAV patients and DPPs than anti-GBM patients, which was in agreement with our study findings (7).

The level of serum creatinine of DPPs at the time of diagnosis was higher than the MPO-AAV group, which might explain why the renal outcome of DPPs was worse than MPO-ANCA positive patients. According to previous studies, other than kidney damage, DPPs exhibited multiple organ involvement contrary to anti-GBM patients (19, 20). It was found that 75% DPPs simultaneously had pulmonary damage and 30% of DPPs displayed cardiovascular system involvement in our study. Patients with anti-GBM disease can produce IgG antibodies targeting the α-3-chain of type IV collagen (α3(IV)NC1), which facilitates the activation of the classical complement pathway inducing neutrophil-mediated inflammation. α3(IV)NC1 has a narrow distribution range in the human body, situated in the basement membrane of glomeruli, alveoli, brain, eye and inner ear (10). However, AAV mainly damages the endothelium of small and medium-sized blood vessels and can involve almost all systems of the whole body (21). The latest Japanese study of Yuka Nishibata et al has suggested that the release of ANCA can damage the kidney and then result in revealing α3(IV)NC1, leading to infiltration of CD11c^+^ macrophages, subsequently the exposed GBM epitope can induce the formation of anti-GBM antibodies (22, 23), which can explain why DPPs have similar characteristics to anti-GBM disease. Therefore, wider organ involvement can be observed in DPPs compared with anti-GBM disease.

With respect to the renal histopathological features, the patients in the DPPs group and anti-GBM group both have crescents in more than half of their glomeruli. Meanwhile, Bowman’s capsule rupture was more frequent in the DPPs group. The research of Samy Hakroush et al. had shown that Bowman’s capsule rupture was frequent in ANCA-GN and independent of glomerular lesions, tubular atrophy and fibrosis (24). Anqun Chen et al. reported that the Bowman’s capsule can protect the visceral epithelial cells from attack by CD8^+^ T cells in crescentic glomerulonephritis (25). Previous studies indicated that the fibrous strand-strengthened membrane of the Bowman’s capsule might act as a barrier preventing invasion of inflammatory cells. When the capsule integrity was destroyed, inflammatory cells can enter the glomerular space more easily and then cause tubulointerstitial damage (26).

Concerning the probability of renal failure, our data are in accordance with previous work showing that DPPs usually had severe renal dysfunction and required dialysis at presentation (5). Accordingly, it was reasonable that DPPs had a higher incidence of ESRD than the single-positive MPO-ANCA group.

Patients in our center received standard immunosuppressive regimens including agents such as glucocorticoids, cyclophosphamide and intravenous methylprednisolone pulse therapy. But there were no patients who received rituximab (RTX) treatment in our cohort. Previous studies shown that autoantibodies and T cell dysregulation were at the core of the pathogenesis for both AAV and anti-GBM disease (27–29). While RTX targeting CD20 has been approved to be effective for patients with AAV and anti-GBM disease, the anti-CD52 monoclonal antibody targeting T cells may also be a potential therapy for DPPs in the future (30–32). In addition, complement blockade through anti-C5 monoclonal antibody and inhibitor of the C5a receptor may also be effective for DPPs (33, 34).

In addition to being an indicator of inflammatory response, we found that the low lymphocyte count could predict the renal outcome of DPPs. Innate immunity plays an important role in AAV. Neutrophils are considered as predominant in this process, at the same time, T cells and B cells are also indispensable. The underlying reasons for that low lymphocyte count can predict the poor renal prognosis in this study are not clear. Lymphocytes contain different subsets of T cells and B cells, some of which promote or inhibit the inflammatory reaction. Further studies are needed to explore the detailed phenotype, proportion and function of these lymphocytes. We assume that one probable reason is that patients with lower lymphocyte count might have higher proportions of non-exhausted autoreactive T cells which drive kidney injury in patients with DPPs, as a previous study has shown that CD8^+^ T cell exhaustion could predict a favorable outcome in multi-system autoimmune and inflammatory diseases such as AAV (35).

It was not surprising that increased serum creatinine and reduced eGFR can be considered as risk factors of renal survival. Samy Hakroush and his team confirmed that Bowman’s capsule rupture was associated with renal survival in ANCA-GN probably related to tubulointerstitial inflammation (24). In addition, rupture of the Bowman’s capsule can facilitate the progression of cellular cresents (36). However, no association has been found between the rupture ratio of the Bowman’s capsule and any pathological index with renal or overall survival condition in our DPPs cohort, perhaps in part owing to the small number of our DPPs patients.

There were several limitations in our study. First, the nature of the retrospective study itself could not fully avoid information and recall bias. Second, the number of DPPs cases we collected was relatively small. Third, not all of DPPs underwent renal biopsy. One reason for the relatively low rate of renal biopsy in the DPPs group were that some patients with severe disease had not received a renal biopsy. Furthermore, during the long follow-up period, some patients dropped out which may partially affect the accuracy of prognosis analysis.

In summary, our work suggested that DPPs mainly affected older persons and usually caused multi-organ injury. Bowman’s capsule rupture was seen more frequently in DPPs. Serological DPPs had a worse renal and patient survival than MPO-AAV patients. We recommend to test for both autoantibodies for all patients with AAV and/or anti-GBM disease. Low lymphocyte count, reduced eGFR and elevated serum creatinine were independent risk factors for renal survival in DPPs. Further studies with a large cohort of patients are required to validate these results.

## Data availability statement

The original contributions presented in the study are included in the article/**Supplementary Material**. Further inquiries can be directed to the corresponding authors.

## Ethics statement

The studies involving human participants were reviewed and approved by Medical Ethics Committee of the Xiangya Hospital of Central South University. The patients/participants provided their written informed consent to participate in this study.

## Author contributions

RT, YZ, XX and HL contributed to the conception of the study. XH, RT, YZ, JO and PE performed the analysis with constructive discussions. CS, J-BC, WL analyzed and interpreted the data. WL, XH, JX, NL analyzed the pathological analysis. RT, Y-OZ, TM, YG and QX contributed significantly to the patient enrollment and follow-up. XH finished the manuscript. RT and YZ supervised and edited the manuscript. JO and PE edited the manuscript. All authors read and approved the submitted version.

## Funding

This work was funded by the National Key Research and Development Program of China (2020YFC2005000 to XX), the Key Research and Development Program of Hunan province (2020WK2008 to YZ), the Natural Science Foundation of Hunan Province (2022JJ30070 to RT, 2021JJ31130 to YZ, and 2020JJ6109 to CS), the Science and Technology Innovation Program of Hunan Province (2020RC5002 to JO), “the Project of Health Commission of Hunan Province (A202303050036 to YZ)”, “YiluqihangShenmingyuanyang” Medical Development and Scientific Research Fund Project on Kidney Diseases (SMYY20220301001 to YZ).

## Acknowledgments

The authors thank all the staff of the Department of Nephrology and the nursing staff for their dedicated assistance in patient follow-up data collection.

## Conflict of interest

The authors declare that the research was conducted in the absence of any commercial or financial relationships that could be construed as a potential conflict of interest.

## Publisher’s note

All claims expressed in this article are solely those of the authors and do not necessarily represent those of their affiliated organizations, or those of the publisher, the editors and the reviewers. Any product that may be evaluated in this article, or claim that may be made by its manufacturer, is not guaranteed or endorsed by the publisher.
