# Supplementary material for: Clinical features and prognosis of MPO-ANCA and anti-GBM double-seropositive patients
Source: Front Immunol. 2022 Oct 27;13:991469. doi: 10.3389/fimmu.2022.991469 (PMC9648717; doi:10.3389/fimmu.2022.991469)
Supplement: Supplementary file 1 [file Table_1.docx]

| **Supplementary Table 1 \|** Baseline clinical characteristics of patients with a renal biopsy. | | | | |
| --- | --- | --- | --- | --- |
|  | **MPO-AAV**  **(n=109)** | **DPPs**  **(n=10)** | **Anti-GBM**  **(n=23)** | ***p* value** |
| Age (year) (mean, SD) | 54.17±16.37 | 63.20±9.66 | 41.78±14.55^a,b^ | <0.0001 |
| Neutrophil (10^9^/L) (mean, SD) | 7.03±4.62 | 6.76±1.78 | 7.29±3.85 | 0.944 |
| Lymphocyte (10^9^/L) (mean, SD) | 1.45±1.55 | 1.08±0.47 | 1.31±0.68 | 0.688 |
| Hemoglobin (g/L) (mean, SD) | 81.61±17.20 | 79.80±9.52 | 81.04±19.55 | 0.946 |
| Platelet (10^9^/L) (mean, SD) | 256.38±106.57 | 231.40±119.78 | 252.52±106.51 | 0.779 |
| Serum albumin (g/L) (mean, SD) | 39.71±14.24 | 40.21±16.14 | 27.57±5.05^a,b^ | 0.001 |
| Serum globulin (g/L) (mean, SD) | 31.97±6.63 | 33.12±7.76 | 27.76±6.88^a,b^ | 0.019 |
| Urinary protein (g/24 h) (median, IQR) | 1.53 (0.85,3.70) | 1.03 (0.50,3.66) | 3.06 (1.26,5.00) | 0.197 |
| Serum creatinine (μmol/L) (mean, SD) | 374.88±251.15 | 456.10±168.35 | 826.29±403.70^a,b^ | <0.0001 |
| eGFR (ml/min per 1.73 m^2^) (median, IQR) | 18.02 (8.50,27.77) | 8.58 (7.77,19.10) | 5.39 (3.82,13.13)^a^ | <0.0001 |
| CRP (mg/dL) (median, IQR) | 16.20 (4.99,41.80) | 13.00(5.13,39.90) | 31.75 (5.86,110.00) | 0.279 |
| ESR (mm/h) (median, IQR) | 71 (42,110) | 86(36.5,120) | 41(20,100) | 0.086 |
| Serum immunological indexes |  |  |  |  |
| sC3 (mg/L) (mean, SD) | 819.92±243.45 | 766.60±195.68 | 896.09±273.21 | 0.289 |
| sC4 (mg/L) (mean, SD) | 264.65±110.07 | 217.65±62.26 | 277.65±96.20 | 0.316 |
| sIgA (mg/L) (mean, SD) | 2674.11±1407.03 | 2332.10±1363.03 | 2229.87±1378.32 | 0.331 |
| sIgG (g/L) (mean, SD) | 14.67±4.79 | 14.03±5.73 | 9.49±4.63^a,b^ | <0.0001 |
| sIgM (mg/L)(mean, SD) | 1121.20±659.87 | 942.70±236.83 | 975.00±531.31 | 0.45 |
| Organ involvement |  |  |  |  |
| Kidney, n% | 109 (100.00) | 10 (100.00) | 23 (100.00) | 1.000 |
| Pulmonary, n% | 60 (55.05) | 6 (60.00) | 10 (43.48) | 0.548 |
| Cardiovascular, n% | 14 (12.84) | 3 (30.00) | 0(0.00)^b^ | 0.032 |
| Nervous system, n% | 13 (11.93) | 0 (0.00) | 0(0.00) | 0.147 |
| BVAS (mean, SD) | 16.31±5.75 | 16.50±7.52 | - | 0.922 |
| ESRD, n (%) | 37 (33.94) | 8 (80.00)^a^ | 19 (82.61)^a^ | <0.0001 |
| Mortality, n (%) | 9 (8.26) | 2 (20.00) | 2 (8.70) | 0.434 |
| eGFR, estimated glomerular filtration rate; CRP, C-reactive protein; ESR, erythrocyte sedimentation rate; BVAS, birmingham vasculitis activity score; ESRD, end stage renal disease; ^a^*p* < 0.05 vs. MPO-AAV and ^b^*p* < 0.05 vs. DPPs. | | | | |
