# Supplementary material for: Clinical features and prognosis of MPO-ANCA and anti-GBM double-seropositive patients
Source: Front Immunol. 2022 Oct 27;13:991469. doi: 10.3389/fimmu.2022.991469 (PMC9648717; doi:10.3389/fimmu.2022.991469)
Supplement: Supplementary file 2 [file Table_2.docx]

| **Supplementary Table 2 \|**  Treatment regimens for patients. | | | | |
| --- | --- | --- | --- | --- |
|  | **MPO-AAV**  **(n=109)** | **DPPs**  **(n=20)** | **Anti-GBM**  **(n=23)** | ***p* value** |
| Corticosteroids, n% | 100 (91.7%) | 20 (100.0%) | 21 (91.3%) | 0.597 |
| Methylprednisolone pulse, n% | 24 (22.0%) | 7 (35.0%) | 11 (47.8%)^a^ | 0.033 |
| Cyclophosphamide, n% | 82 (75.2%) | 15 (75.0%) | 17 (73.9%) | 1.000 |
| Plasma exchange, n% | 34 (31.2%) | 13 (65.0%)^a^ | 18 (78.3%)^a^ | ＜0.001 |
| ^a^ *p* < 0.05 vs. MPO-AAV. | | | | |
