# Supplementary figures and images for: Clinical features and prognosis of MPO-ANCA and anti-GBM double-seropositive patients
Source: Front Immunol. 2022 Oct 27;13:991469. doi: 10.3389/fimmu.2022.991469 (PMC9648717; doi:10.3389/fimmu.2022.991469)

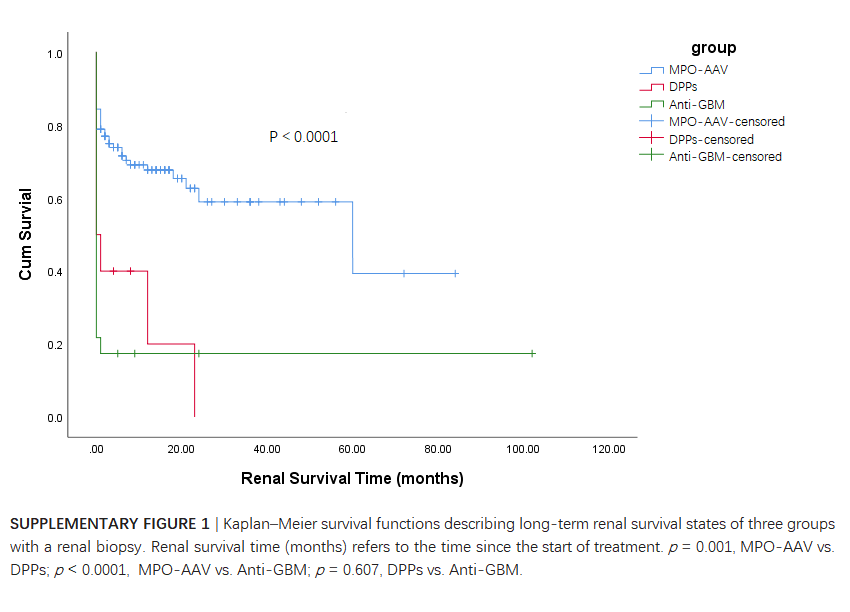

Supplement: Supplementary file 3 [file Image_1.tif]

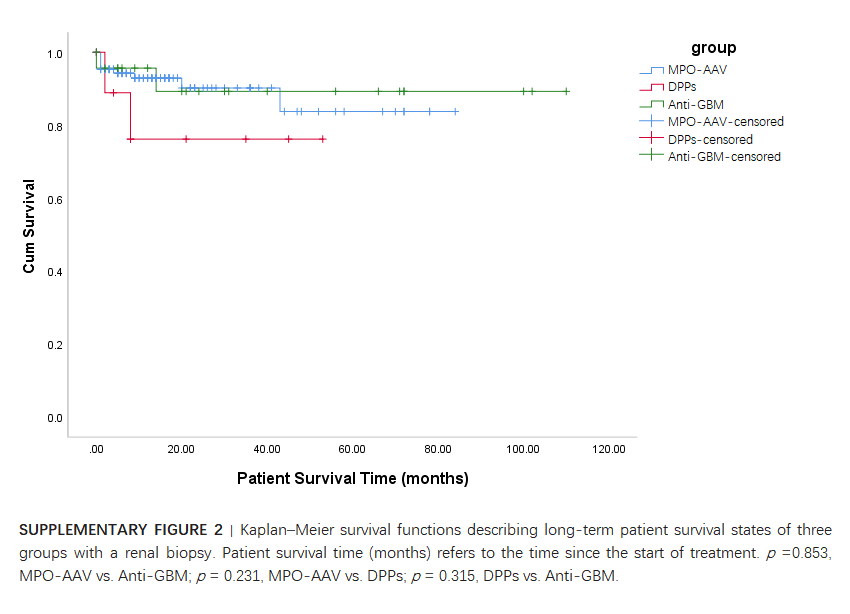

Supplement: Supplementary file 4 [file Image_2.tif]
